# Anesthetic management of Stanford type B acute aortic dissection that occurred during transcatheter aortic valve implantation under monitored anesthesia care: A case report

**DOI:** 10.1016/j.heliyon.2023.e21278

**Published:** 2023-10-20

**Authors:** Shusuke Okamoto, Takuya Okada, Norihiko Obata, Yu Yamane, Koichiro Masada, Masahiko Iseki, Masaharu Nagae

**Affiliations:** a Department of Anesthesiology and Pain Clinic, Hyogo Prefectural Harima-Himeji General Medical Center, 3-264 Kamiya-cho, Himeji, 670-8560, Japan; b Division of Anesthesiology, Department of Surgery Related, Kobe University Graduate School of Medicine, 7-5-2 Kusunoki-cho, Chuo-ku, Kobe, 650-0017, Japan

**Keywords:** Transcatheter aortic valve implantation under monitored anesthesia care, Stanford type B acute aortic dissection, Aortic stenosis, Extracorporeal membrane oxygenation, Case report

## Abstract

We report a case involving anesthetic management of Stanford type B acute aortic dissection occurred during transcatheter aortic valve implantation (TAVI) under monitored anesthesia care (MAC) in a patient with aortic stenosis (AS). An 87-year-old woman was undergoing TAVI under MAC for severe AS. During the surgery, the patient suddenly moved possibly because of pain. This was followed by hemodynamic collapse. She was then transitioned to general anesthesia, and extracorporeal membrane oxygenation (ECMO) was initiated. Transesophageal echocardiography revealed a Stanford type B acute aortic dissection, which was safely managed perioperatively with appropriate interventions.

## Highlights

• One of the complications of TAVI under MAC is acute aortic dissection.
• Acute aortic dissection (AAD) is a serious and potentially fatal complication.
• Anesthesiologists play a crucial role in managing patients who develop AAD.
• If AAD occurs during TAVI under MAC, switching to general anesthesia should be considered.
• It is important to appropriately manage blood pressure and protect multiple organs.

## 1 Introduction

Transcatheter aortic valve implantation (TAVI) has become the standard treatment for aortic stenosis (AS) in elderly high-risk patients [1,2]. In recent years, the number of cases of TAVI has increased significantly due to the expansion of its indications. One of the complications of TAVI is aortic dissection, which typically has a low incidence rate of 0.2–0.3 % but is a serious and potentially fatal complication with high mortality rates once it occurs [3,4]. While there are very few reports on its anesthetic management, anesthesiologists play a crucial role in managing patients who develop acute aortic dissection during TAVI under monitored anesthesia care (MAC). We report the anesthetic management of Stanford type B acute aortic dissection that occurred during a TAVI under MAC for AS.

## 2 Case presentation

An 87-year-old woman was diagnosed with scleroderma when she was 77 years old. She was under regular monitoring with transthoracic echocardiography for the development of pulmonary hypertension. At 86 years, the patient was diagnosed with moderate aortic stenosis (AS). At 87 years, she had symptoms of exertional dyspnea and was diagnosed with severe AS and worsening pulmonary hypertension, with a tricuspid regurgitation pressure gradient (TRPG) of 66 mmHg. Furthermore, upon performing the right heart catheterization, the mean pulmonary artery pressure was 32 mmHg. She was prescribed diuretics, which resulted in mild symptom improvement. Following review by the heart team, TAVI was considered based on her age, frailty, and high risk of surgical aortic valve replacement. The patient's surgical risk scores indicated a Society of Thoracic Surgeons score of 5.13 %, logistic-Euro score of 30.24 %, frailty score of 4, and New York Heart Association Functional Classification （NYHA） level of Class Ⅲ.

At the time of admission, she was 140.3 cm tall, weighed 28.3 kg, and had a blood pressure of 100/50 mmHg, heart rate of 74 bpm with regular rhythm, respiratory rate of 20 breaths/min, and SpO_2_ of 95 % on room air. Transthoracic echocardiography showed paradoxical low-flow low-gradient severe aortic stenosis with an ejection fraction of 56.9 % (assessed by the modified Simpson's method), aortic valve area of 0.83 cm^2^, peak velocity of 3.4 m/s, and mean pressure gradient of 26.6 mmHg. In addition, the patient had moderate aortic regurgitation, mild-moderate mitral regurgitation, and mild tricuspid regurgitation.

**Fig. 1:**
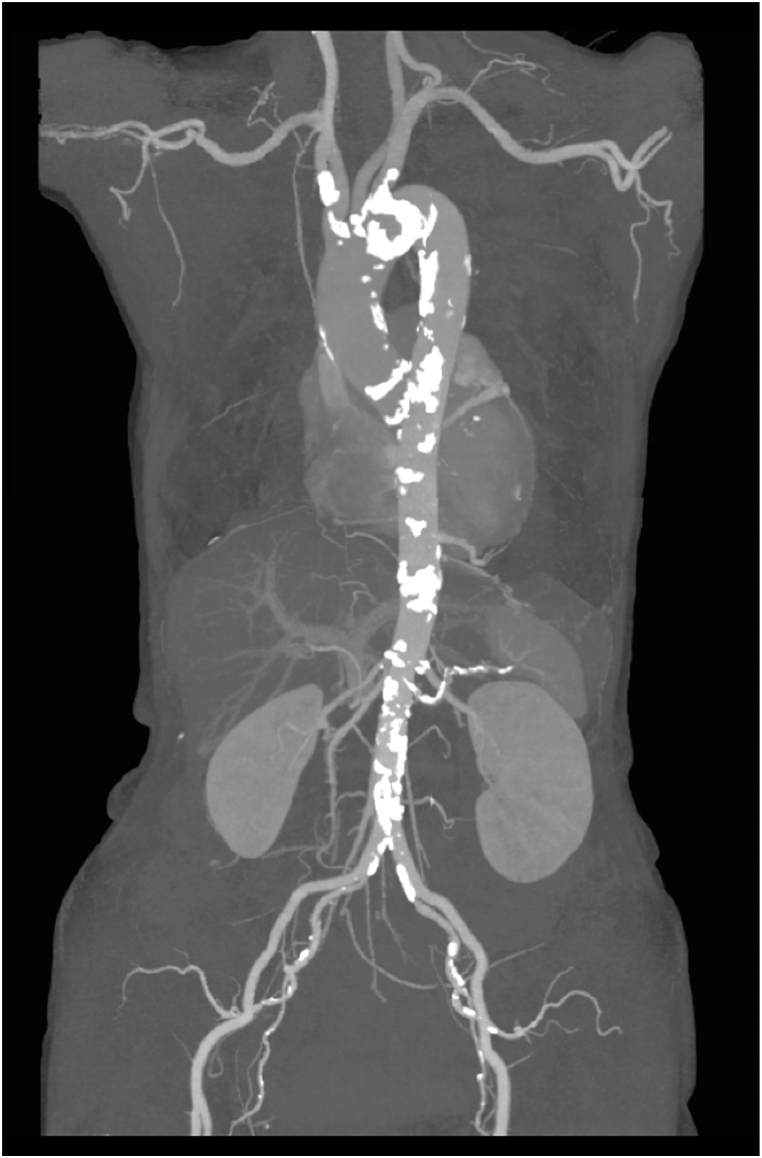
Preoperative computed tomography image of the aorta.

The patient also had the complication of scleroderma, which was suspected as being the cause of the vascular stenosis and calcification observed on contrast-enhanced CT examination of the cervical and lower extremity vessels (Fig. 1). However, after the heart team's evaluation, she was scheduled for TAVI by the transfemoral approach (TF-TAVI), after it was determined that this was feasible.

**Fig. 2:**
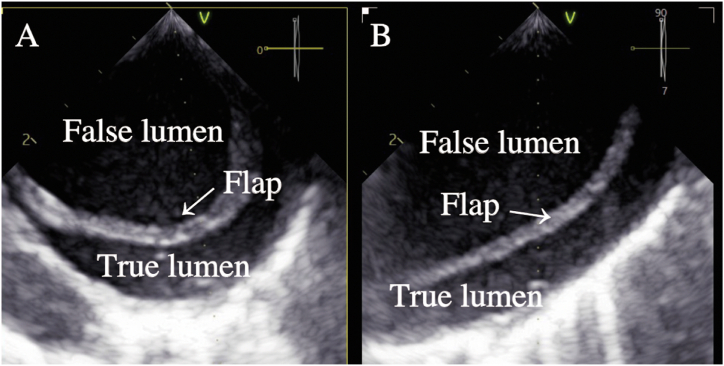
Transesophageal echocardiography images demonstrated dissection of the descending aorta and flap formation at the distal to the left subclavian artery. (A) Short-axis view of the descending aorta. (B) Long-axis view of the descending aorta. The true lumen was compressed by the false lumen.

**Fig. 3:**
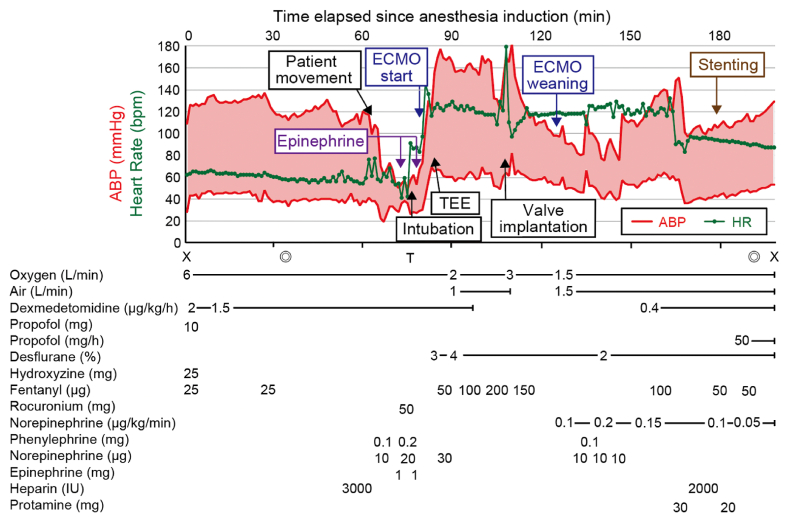
Anesthesia record. ECMO, extracorporeal membrane oxygenation; TEE, Transesophageal echocardiography; X, start and end of anesthesia; ◎, start and end of operation; T, intubation; ABP, arterial blood pressure; HR, heart rate.

**Fig. 4:**
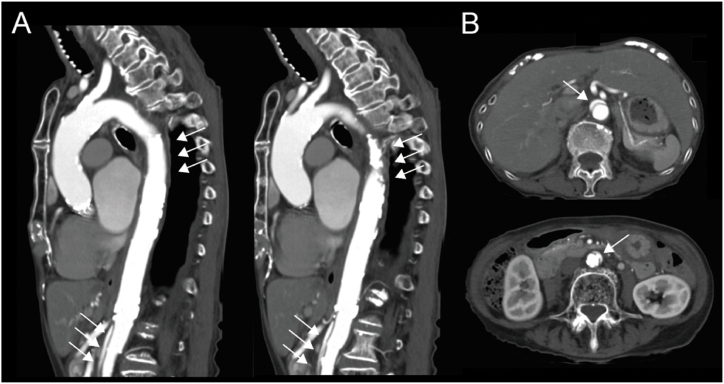
Postoperative computed tomography images of the aorta. (A) Sagittal plane of the aorta. Aortic dissection was observed from the distal arch to the left common iliac artery. (B) Axial plane of the aorta. The false lumen was compressed by the true lumen.

We planned to conduct this case using local anesthesia with sedation, and used a monitor that included an electrocardiogram, transcutaneous oxygen saturation, non-invasive blood pressure measurement, invasive arterial pressure measurement (right radial artery), electroencephalogram (BIS®, Covidien Japan Inc., Tokyo, Japan), and cerebral oximeter (INVOS®, Covidien Japan Inc., Tokyo, Japan). The patient was sedated with intravenous fentanyl 25 μg, propofol 10 mg, and hydroxyzine 25 mg, and sedation was maintained with continuous intravenous infusion of dexmedetomidine 1.5–2 μg/kg/hr, under invasive arterial pressure monitoring. After the patient reached an adequate level of sedation, surgeons obtained access to the right and left femoral arteries under echocardiographic guidance and local anesthesia. An 8 Fr sheath was inserted into the right femoral artery, and 5 Fr sheaths were inserted into the left femoral artery and right femoral vein. Next, surgeons inserted a temporary pacemaker (Zeon Temporary Pacing Catheter A®, Zeon Medical Inc., Tokyo, Japan) via the right femoral vein, positioning it at the apex of the right ventricle. A pigtail catheter was inserted from the right femoral artery to the ascending aorta, and a 14 Fr eSheath (Edwards Lifesciences Inc., Irvine, CA, USA) was inserted over a guidewire (Egoist®, Interventional Guide Wire, Medico's Hirata Inc., Osaka, Japan). There was no resistance during insertion of the eSheath. After inserting the guidewire (Safari Pre-shaped TAVI Guidewire®, Boston Scientific Co, Marlborough, USA) from the left femoral artery into the left ventricle, surgeons attempted inserting a 23 mm artificial valve delivery system (Edwards Commander Transfemoral Delivery System®, Edwards Lifesciences Inc.). Although insertion was possible, there was strong resistance, and the patient suddenly moved possibly because of pain. Thereafter, the patient's systolic blood pressure immediately decreased from around 100 mmHg–70 mmHg, and her heart rate slowed to 40 bpm, resulting in the need for relying on the backup pacemaker. There was a poor response to vasopressors, such as epinephrine, although it was confirmed that there was no acidosis due to high CO_2_ levels on the blood gas analysis, and no significant stenosis on coronary angiography. Her systolic blood pressure remained at approximately 50–60 mmHg and did not improve. Therefore, we decided to switch to general anesthesia and initiate extracorporeal membrane oxygenation (ECMO). After administering rocuronium 50 mg and performing endotracheal intubation, surgeons secured the left femoral vein and established veno-arterial ECMO (Mera Excelline circuit HP2®, Senko Medical Ins., Tokyo, Japan) via the left femoral artery (Capiox® 15 Fr, percutaneous catheter, Terumo Co, Tokyo, Japan) and left femoral vein (Capiox® 21 Fr). General anesthesia was maintained with the inhaled anesthetic agent, 3–4 % desflurane, and fentanyl administered as needed while monitoring the blood pressure. After the initiation of ECMO, the patient's blood pressure stabilized quickly. Transesophageal echocardiography (TEE) revealed aortic dissection in the descending aorta. The dissection did not extend to the ascending aorta, and a flap was observed beyond the distal arch of the aorta, with the true lumen being compressed by the false lumen (Fig. 2A and B). Since the patient's hemodynamics were stable and the dissection did not extend to the ascending aorta, the surgery was continued. Under TEE observation of the aorta, the artificial valve (SAPIEN 3® 20 mm, Edwards Lifesciences Inc.) was carefully advanced and placed under rapid pacing (180 bpm). After valve placement, the patient's hemodynamics rapidly improved, making it possible for her to be successfully weaned off ECMO. After weaned off ECMO, blood pressure was maintained with continuous infusion of norepinephrine and single dose of phenylephrine and norepinephrine. Thereafter, aortic angiography was performed, which revealed no damage in the ascending aorta, although aortic dissection was observed in the distal arch. Additionally, due to compression of the right renal artery by the false lumen, a stent (Express SD®, Boston Scientific Co.) was placed at the origin of the renal artery. Finally, since lower extremity angiography revealed no abnormalities, the surgery was concluded. The entire operation lasted for 168 minutes (Fig. 3). Postoperatively, the patient was sedated with propofol, remained intubated, and transferred to the ICU. The postoperative CT scan showed aortic dissection from the distal arch to the left common iliac artery, although the false lumen was now compressed by the true lumen (Fig. 4A and B). Based on her clinical course, we suspected that the aortic intima had been damaged during the insertion of the artificial valve delivery system.

Postoperatively, we managed the patient conservatively using blood pressure-lowering medication (nicardipine, etc.) as recommended for Stanford type B acute aortic dissection. Her postoperative course was uneventful, and her trachea was extubated on the first postoperative day, and she was discharged on the 23rd postoperative day. The patient's NYHA class improved from class III before the surgery to II at the time of discharge, and she was able to walk slowly. Transthoracic echocardiography at the time of discharge showed improvement in the aortic valve area to 1.68 cm^2^, with a peak flow velocity of 1.76 m/s, mean pressure gradient of 6.0 mmHg and TRPG of 38 mmHg, indicating improvement in AS and pulmonary hypertension.

## 3 Discussion

There are very few reports on the anesthetic management of patients who develop acute aortic dissection during TAVI under MAC. In this report, we provided a detailed description of the anesthetic management of a patient with Stanford type B acute aortic dissection that occurred during TAVI under MAC.

The incidence of aortic dissection as a complication of TAVI is reportedly 0.2–0.3 % [3,4]. Although it is a rare complication, it is a serious condition requiring appropriate management. Most reported cases are Stanford type A acute aortic dissection [[4], [5], [6], [7]], and there are very few reports of Stanford type B acute aortic dissection [[8], [9], [10]].

There are several mechanisms by which aortic dissection can occur during TAVI. The first involves the mechanical stress caused by catheter insertion. During TAVI, a catheter is used to deliver an artificial valve into the aorta, which can cause mechanical stress on the aortic wall and potentially cause injury. Second, is the mechanical stress caused by valve placement. Artificial valve insertion leads to strong forces being applied on the aortic wall, which can cause compression and potentially lead to aortic dissection, depending on the location, size, and shape of the valve. Finally, balloon expansion can also contribute to aortic dissection. After valve placement, a balloon is sometimes used to expand the valve further, which also exerts strong pressure on the aortic wall, with the potential to cause aortic dissection. These factors can together precipitate aortic dissection [4]. In this case, it is thought that the mechanical stress from device insertion via the left femoral artery might have damaged the aortic intima.

When aortic dissection occurs during TAVI, if it is a Stanford type A acute aortic dissection, emergency ascending aortic replacement or aortic arch replacement should be considered to rescue the patients. However, the risk of these procedures is very high in patients in whom TAVI was primarily selected instead of surgical aortic valve replacement. On the other hand, there are only few case reports of Stanford type B acute aortic dissection occurring during TAVI, and no treatment guidelines are available. Hence, it is considered appropriate to follow the treatment approach for naturally occurring Stanford type B acute aortic dissection. As previously reported, in cases with complications such as aortic rupture or organ ischemia, invasive treatments such as thoracic endovascular aortic repair (TEVAR) are considered [8,9], while conservative treatment is recommended in the absence of complications [10]. The treatment strategy should be carefully considered by a heart team based on the patient's background and clinical status.

Regarding anesthesia methods for TAVI, local anesthesia has some advantages, such as shortening of procedure time and the duration of hospitalization, along with reduction in the amount of vasopressor administered during surgery, although general anesthesia also has some advantages, such as the ease of use of TEE, which enables performance of TEE in real-time, allowing detailed evaluation of paravalvular leakage [11]. Since 2014, several meta-analyses have been conducted comparing local and general anesthesia for TAVI, but until the analysis conducted in 2017, no significant differences were found in the incidence of complications and the prognosis [12,13]. However, a meta-analysis reported in 2018 showed a significant improvement in 30-day mortality rate in the local anesthesia group [14]. In addition, recent large-scale analyses in Europe and the United States have also shown significant improvement in prognosis with local anesthesia, suggesting the superiority of local anesthesia [15,16]. However, since the rate of switching from local anesthesia to general anesthesia during TAVR has been reported to be 3.1 % (0.5–12.0 %) [17], it is necessary to be prepared for the potential transition to general anesthesia when managing cases under local anesthesia.

In cases of acute aortic dissection occurring during TAVI under MAC, it is important that quickly and appropriately manage blood pressure and protect the brain and other organs to prevent symptom progression. In terms of blood pressure management, blood pressure should be controlled with the judicious use of antihypertensive drugs to reduce the load on the aortic wall and prevent the progression of the dissection. However, caution is required in the administration of antihypertensive drugs, avoiding their excessive administration, since this can conversely cause hypotension and organ ischemia. Appropriate pain management is also important because the pain associated with aortic dissection can increase blood pressure and accelerate the progression of dissection. Therefore, if acute aortic dissection occurs during TAVI under MAC, switching to general anesthesia during surgery should be considered. However, changing to general anesthesia carries potential risks, including further destabilization of hemodynamics and an increased risk of respiratory complications due to intubation. These factors could adversely affect the patient's prognosis, so the decision to switch should be made carefully.

In this case, the cause of hemodynamic collapse might have been related to the strong resistance encountered during device insertion via the left femoral artery, which could have led to stimulation of the vagal reflex from pain at the time of catheter insertion or from the pain felt secondary to the type B acute aortic dissection, resulting in prolonged bradycardia and hypotension. In this case, prompt switching to general anesthesia, initiation of ECMO, and the subsequent use of TEE enabled diagnosis of the Stanford type B acute aortic dissection and its subsequent appropriate management.

## 4 Conclusion

We successfully managed a patient with Stanford type B acute aortic dissection that occurred during TAVI under MAC for AS. The possibility of aortic dissection as a serious complication of TAVI under MAC should be remembered.

## Informed consent

Written informed consent was obtained from the patient for publication of this case report.

## Funding statement

This work did not receive any specific grant from funding agencies in the public, commercial, or not-for-profit sectors.

## Data availability statement

Data will be made available on request.

## CRediT authorship contribution statement

**Shusuke Okamoto:** Writing – original draft. **Takuya Okada:** Writing – review & editing, Supervision. **Norihiko Obata:** Supervision. **Yu Yamane:** Writing – original draft, Data curation. **Koichiro Masada:** Data curation, Writing – original draft. **Masahiko Iseki:** Data curation, Writing – original draft. **Masaharu Nagae:** Supervision, Writing – review & editing.

## Declaration of competing interest

The authors declare that they have no known competing financial interests or personal relationships that could have appeared to influence the work reported in this paper.
